# Microbially induced calcium carbonate precipitation through CO_2_ sequestration via an engineered *Bacillus subtilis*

**DOI:** 10.1186/s12934-024-02437-7

**Published:** 2024-06-10

**Authors:** Katie A. Gilmour, Prakriti Sharma Ghimire, Jennifer Wright, Jamie Haystead, Martyn Dade-Robertson, Meng Zhang, Paul James

**Affiliations:** 1 Living Construction Group, Hub for Biotechnology in the Built Environment, Department of Applied Sciences, Northumbria University at Newcastle, Newcastle, NE1 8ST UK; 2 Hub for Biotechnology in the Built Environment, School of Architecture, Planning and Landscape, Newcastle University, Newcastle, NE1 7RU UK; 3 Diosynth Biotechnologies, FUJIFILM, Billingham, TS23 1LH UK; 4 Living Construction Group, Hub for Biotechnology in the Built Environment, Department of Architecture and Built Environment, Northumbria University at Newcastle, Newcastle, NE1 8ST UK

**Keywords:** Carbon capture, MICP, Carbon sequestration, Carbonic anhydrase, Calcite, Recombinant protein expression

## Abstract

**Background:**

Microbially induced calcium carbonate precipitation has been extensively researched for geoengineering applications as well as diverse uses within the built environment. Bacteria play a crucial role in producing calcium carbonate minerals, via enzymes including carbonic anhydrase—an enzyme with the capability to hydrolyse CO_2_, commonly employed in carbon capture systems. This study describes previously uncharacterised carbonic anhydrase enzyme sequences capable of sequestering CO2 and subsequentially generating CaCO_3_ biominerals and suggests a route to produce carbon negative cementitious materials for the construction industry.

**Results:**

Here, *Bacillus subtilis* was engineered to recombinantly express previously uncharacterised carbonic anhydrase enzymes from *Bacillus megaterium* and used as a whole cell catalyst allowing this novel bacterium to sequester CO_2_ and convert it to calcium carbonate. A significant decrease in CO_2_ was observed from 3800 PPM to 820 PPM upon induction of carbonic anhydrase and minerals recovered from these experiments were identified as calcite and vaterite using X-ray diffraction. Further experiments mixed the use of this enzyme (as a cell free extract) with *Sporosarcina pasteurii* to increase mineral production whilst maintaining a comparable level of CO_2_ sequestration.

**Conclusion:**

Recombinantly produced carbonic anhydrase successfully sequestered CO_2_ and converted it into calcium carbonate minerals using an engineered microbial system. Through this approach, a process to manufacture cementitious materials with carbon sequestration ability could be developed.

## Introduction

Concrete is one of the most widely used building materials due to its low-cost production and long-term durability. However, each ton of cement produced generates 0.6 t CO_2_, with current global production at 4.3 million tons per annum and predicted to increase [1]. Therefore, a more environmentally sustainable alternative is needed to replace concrete or reimagine the process by which cementations materials are made.

One alternative method to generate cementitious material may be through microbial processes, and indeed such approaches have generated interest from academic and industrial communities. Microbially induced calcium carbonate precipitation (MICP) is a well-studied process by which microbes influence the biomineralisation of calcium carbonates [2]. Current applications include self-healing concrete, soil bioconsolidation, and soil remediation. Biologically, this process has been shown to occur outside of cells using bacteria cells as a nucleation point [3, 4], or internally as shown in cyanobacteria [5]. The process by which this occurs varies depending on the microbe involved, but classically, the urease pathway in S*porosarcina pasteurii* is considered as a model for this process [6]. Through urea hydrolysis (catalysed by urease), ammonia and carbamate are formed (Eq. 1), a further series of spontaneous reactions then occur (Eqs. 2–4), resulting in a local pH increase. This environmental change, coupled with the negatively charged cell surface acts to attract positively charged calcium ions, allows calcium carbonate formation and precipitation (Eq. 5) [7].

CO(NH_2_)_2_ + H_2_O → NH_2_COOH + NH_3_ (1).

NH_2_COOH + H_2_O → NH_3_ + H_2_CO_3_ (2).

2NH_3_ + 2H_2_O → 2NH_4_^+^ + 2OH^−^ (3).

2OH^−^ + H_2_CO_3_ → CO_3_^2−^ + 2H_2_O (4).

Ca^2+^ + CO_3_^2−^ → CaCO_3_ (5).

However, another pathway, carbonic anhydrase (CA), has been implicated in MICP and CO_2_ sequestration (Eq. 6). CAs catalyse the reversible hydration of CO2 to HCO_3_^−^ and a proton through a 2-stage ping-pong mechanism utilising a zinc bound hydroxide ion found in the enzymes active site. Under alkaline conditions, HCO_3_^−^ can interact with calcium ions and be precipitated as calcium carbonate. Unlike urease, in which high levels of ammonia are produced, MICP via CA does not produce the same harmful by-products.

H_2_O + CO_2_ ↔ HCO_3_^−^ + H^+^ (6).

The use of this enzyme could provide a sustainable approach to produce cementitious material whilst also acting as a carbon dioxide sequestrator and storage system, removing atmospheric CO_2_. Although carbon capture can occur abiotically, and this reaction is thermodynamically favourable, without CA to act as a catalyst, the formation of hydrogen carbonate ions is very slow [8]. Research into how to use CAs for carbon capture has attracted attention with CO_2_ Solutions (Canada) [9], Novozymes [10], EPRI (Electric Power Research Institute) of America [11] and Carbozyme (United States) [12] testing various purified CAs for use in high CO_2_ output areas (e.g. steel works sites) as a viable and sustainable carbon capture system. However, the CAs used by these industrial ventures have largely originated from archaea or eukaryotic organisms, because of the need for them to be thermophilic.

CAs are also present in many bacterial species, including those commonly found in marine and soil environments. There are several classes of CA, with the most common in bacteria being α-, β-, and ζ-CAs classes and these have a central zinc ion surrounded by three histidine residues and a water molecule/hydroxide ion [13]. The structure of an α-CA from a thermophilic marine bacterium, *Thermovibrio ammonificans* was previously characterized and the activity of the purified enzyme was found to have high CO_2_ hydrolysis activity [14]. Other bacteria with CAs include *Neisseria gonorrhoeae* which was successfully recombinantly expressed in *Escherichia coli* [15] and *Bacillus megaterium*, a gram-positive aerobic bacterium which possesses both a urease and CA pathway [16]. The appearance of both these enzymes may indicate a synergistic relationship, however, CAs from *B. megaterium* have not been fully studied or characterized and the urease pathway is considered poor compared to model organisms such as *S. pasteurii* [17], which as a bacterium is not yet possible to engineer. However, as *B. megaterium* commonly occurs in soil, it would be a good candidate for potential biocementation applications. The ideal engineered biocementation bacteria will be able to express CA enzymes at high levels and at a higher rate when compared to the WT organisms and the expression of CA can be induced to control when the process is “switched on”.

In this study, we genetically modified *Bacillus subtilis* by incorporating and over-expressing CAs sourced from a diverse array of soil microorganisms. Our objective was to cultivate novel strains with improved MICP process and enhanced capability for CO_2_ sequestration, thereby creating carbon negative cementitious materials that could contribute to carbon negative sustainability goals. After screening six CAs initially, we pinpointed two promising CAs from *B. megaterium*. We then assessed their ability to hydrate CO_2_ when expressed recombinantly in *B. subtilis*. Through further exploration of MICP using these engineered *B. subtilis* strains we discovered their capacity to efficiently sequester CO_2_ and subsequently store carbon via the formation of calcium carbonate crystals.

## Materials & methods

### Materials

Restriction endonucleases and DNA-modifying enzymes were obtained from ThermoFisher Scientific (UK). All oligonucleotides were obtained from Integrated DNA Technologies (USA). Plasmid pHT253 was obtained from MoBiTec (DE). All other reagents were obtained from Fisher (UK) unless otherwise stated.

### Strains, constructs, and DNA assembly

The protein sequence of *Thermovibrio ammonificans* a-CA (TaCA: WP_013538320.1), a well-characterised CA [14], was used as a query in a BLASTP search against the *B. megaterium* protein database to identify two b-CA sequences (b-CA_ytiB: WP_116074043 and b-CA_yvdA: WP_168241007), additionally searches were carried out on *B. subtilis* (b-CA_ytiB: WP_003229074 and b-CA_yvdA: WP_010886620) and *S. pasteurii* (β-CA: WP_115363014 and γ-CA: WP_115362601). These CAs were then synthesized as g-blocks by IDT and the DNA coding for them and TaCA were cloned into Pgrac100 promotor pHT253 using cut sites BamH1 and Xho1 to produce pHT253_TaCAa, pHT253_BmCAb_ytiB, pHT253_BmCAb_yvdA, pHT253_BsCA_ytib, pHT253CA_yvda, pHT253_SpCAb and pHT253_SpCAg – now referred to as TaCA, BmCAb1, BmCAb2, BsCA1, BsCA2, SpCAb, and SpCAg, respectively. Plasmids were transformed into *E. coli* Top10 cells for verification and propagation, and then transformed into *B. subtilis* strain 168 (BS168 hereafter) for expression using a method adapted from Anagnostopoulos and Spizizen [18]. Constructs were verified by Sanger sequencing (Genewiz, UK). All protein sequences are provided in the supplementary materials. An empty vector (EV) (pHT253) was also transformed into *B. subtilis*, as the control for enzyme activity. *S. pasteurii* DSM33 was used as a control model organism for MICP, and *B. megaterium* DSM32 was used in these experiments to simulate natural expression of urease and CA.

### Culture media

All engineered *E. coli* were grown in Luria Broth (LB) media with 50 ng/mL ampicillin. For protein expression experiments, *B. subtilis* was grown in 2X yeast extract tryptone (2YT) media comprising of 16 g/L tryptone, 10 g/L yeast extract, 5 g/L NaCl, supplemented with 16 ng/mL chloramphenicol and all the media was adjusted to pH 7. Overnight cultures of *S. pasteurii* were grown in 3 g/L nutrient broth with 20 g/L urea, and *B. megaterium* cultures were grown in 3 g/L nutrient broth only.

### Recombinant protein expression

To overexpress recombinant CA, BS168 transformants were grown in 10 mL of 2YT with 17 µg/mL chloramphenicol at 37 °C for 16 h. Starter cultures were then used to inoculate 500 mL of fresh 2YT + chloramphenicol at ratio of 3:50 and growth until OD_600nm_ reached 1. Protein expression was then induced by addition of 1 mM isopropyl-β-d-1-thiogalactopyranoside (IPTG) and the culture was incubated at 20–30 °C for 20 h at 100 RPM. Cells were then harvested by centrifugation at 5000 ×g at 4 °C for 15 min and resuspended in 40 mL of buffer A (100 mM Tris, 0.5 mM NaCl and 20 mM imidazole, pH 7.4).

Cells were lysed by sonication (QSonica ultrasonic processor, QSonica, USA) with addition of 0.1% (w/v) lysozyme (from chicken egg, Fluka Analytical, UK) at amplitude 30% (40 μm) at 30 s intervals with 30 s cooling off periods for 8 cycles. This crude lysate was pelleted by centrifugation at 20,000 ×g at 4 °C for 20 min. The supernatant containing cell free extract (CFE) was then collected and concentration was calculated using a Bradford assay with 12 standards using NanoDrop One (ThermoScientific, UK). CFE was stored in 50% glycerol in − 80 °C for use in future experiments. Expression was confirmed by visualization on Coomassie blue stained SDS-PAGE gel (supplementary).

### Phenol red assay

To measure enzyme activity in hydration of CO_2_, a colorimetric assay was carried out according to James et al. [14]. with some modifications. For each recombinant CA (BmCAb1, BmCAb2, BsCAb1, BsCAb2, SpCAb, SpCAg, TaCA) and control EV, 30 µL of 5 mg/mL CFE were added to 500 µL of phenol red buffer (10 mM HEPESs, 20 mM NaCl, 0.2% (v/v) phenol red, pH 8.3). A saturated CO_2_ solution was prepared by bubbling CO_2_ through 250 mL of sterile dH_2_O for 1 h. 500 µL of saturated CO_2_ was added to the phenol red enzyme mix and the time taken for colour to change from red to orange was recorded. The activity was then recorded as Wilbur-Anderson Units (Eq. 7), where T_0_ is the time taken for EV experiment to change colour, and T is the time taken for the enzymatic experiments to change colour.

7 $$\frac{units}{mg} = \frac{2 \times ({T}_{0}-T)}{T x mg enzyme in reaction mix}$$

### Calcium carbonate production through cells vs. CFE

Overnight cultures of *B. subtilis* with each CA (BmCAb2, BmCAb1 and TaCA) were inoculated into conical flasks containing 50 mL biocementation media (3 g/L nutrient broth, 93.5 mM NH_4_Cl, 500 mM urea, 500 mM CaCl_2_) [19] with chloramphenicol to a starting OD_600_ of 0.2. After 24 h growth at 30 °C, 200 RPM, expression was induced, and the experiment was incubated for a further 4 days in the same condition. Minerals were then collected by centrifugation and washed in phosphate buffered saline (PBS; 8 g/L NaCl, 0.2 g/L KCl, 1.44 g/L Na_2_HPO_4_, 0.24 g/L KH_2_PO_4_; pH 8) and stored for SEM and XRD analysis. In addition to *B. subtilis* with EV, the biomineralization through *S. pasteurii* and *B. megaterium* cells were also set up in the biocementation media without antibiotic as control. The biomineralisation was also investigated using the CFEs (as harvested previously) which were added to give final concentration of 1.5 mg/mL at the same timepoint that whole cell experiments were induced with 1mM IPTG. CFE from EV was used as a control. All experiments were run in triplicate.

### CO_2_ sequestration to form calcium carbonate

A starter culture of *B. subtilis* with each CA (BmCAb2, BmCAb1 and TaCA) was grown at 30 °C, 200 RPM in LB media with chloramphenicol for 16 h and then 200 µL was spread on a 1% agar plate with biomineralization media and chloramphenicol. The expression of the proteins was induced by addition of 1 mM IPTG on each plate. Plates were then incubated for 72 h inside an anaerobic chamber (Coy Lab Products, USA) with a CO_2_ generation sachet (2.5 L, ThermoScientific) at 30 °C. The CO_2_ concentration was measured using a Desktop Indoor Air Quality Datalogger (ExTech CO210, Extech, USA). Additionally, another set of experiments was set-up with 3 × 10^8 ^*S. pasteurii* cells with 200 µL of 5 mg/ml CFE from BmCAb2. The experiments were performed in triplicate and the cells with the EV setting up as the negative control. Positive controls included using *S. pasteurii* and *B. megaterium* in place of *B. subtilis*. The experiment was carried out as described above without antibiotics and nutrient broth media included 20 g/L urea for starter cultures of *S. pasteurii* and nutrient broth without antibiotics for *B. megaterium.*

### Microscopic analyses

Scanning electron microscopy (SEM) was used to investigate the morphology and macrostructure of the minerals produced from biomineralization experiments. Samples were prepared for SEM analysis by drying at room temperature (20 ± 2 °C) and then mounting on an aluminium stub using a carbon sticker and coating with chromium. Samples were then stored in a desiccator before imaging using a TESCAN Mira 3 (TESCAN, CZ) in conjunction with Alicona 3D imaging software (Brucker Alicona, AT).

### X-ray diffraction (XRD) analysis

CaCO_3_ crystals were recovered from liquid and agar plate, where the agar was removed by melting and boiling washing in dH_2_O. Minerals were then air dried at room temperature (20 ± 2 °C) and ground to a fine powder using a pestle and mortar and stored in a desiccator. CaCO_3_ powder was spread uniformly onto a 10 mm × 10 mm sample holder. A Rigaku Ultima XRD analyser was used with CuKα (λ = 1.54056 Å) at 50 kV and 40 mA and RAS files were generated. Results were then analysed with reference to the crystallography open database using PANalytical X’Pert HighScore Plus pattern analysis software (Malvern, UK) with Rietveld refinement [20].

### Statistical analysis

Statistical analysis of results obtained from phenol red assay and CO_2_ sequestration experiments were calculated by running Analysis of Variance (ANOVA) in R Studio [21]. All experiments were performed in biological and technical triplicates (*n* = 9). P-values were obtained following normality checks, and post-hoc Turkey tests were carried out, P-values < 0.05 were determined to be significant. Graphs were then created in GraphPad Prism v10 (GraphPad, USA).

## Results and discussion

### Expression and activity of CAs

Expression of TaCA, BsCAb1, BsCAb2, SpCAg, SpCAb, BmCAb1 and BmCAb2 was achieved in *B. subtilis* at both temperatures tested (20 °C and 30 °C) and was found to be present in CFE, however, they were best expressed at 30 °C as observed on SDS gel (Figure S1) and Bradford assay. Growth of cells for expression or CFE generation was therefore carried out at 30 °C for the rest of the study.

**Fig. 1:**
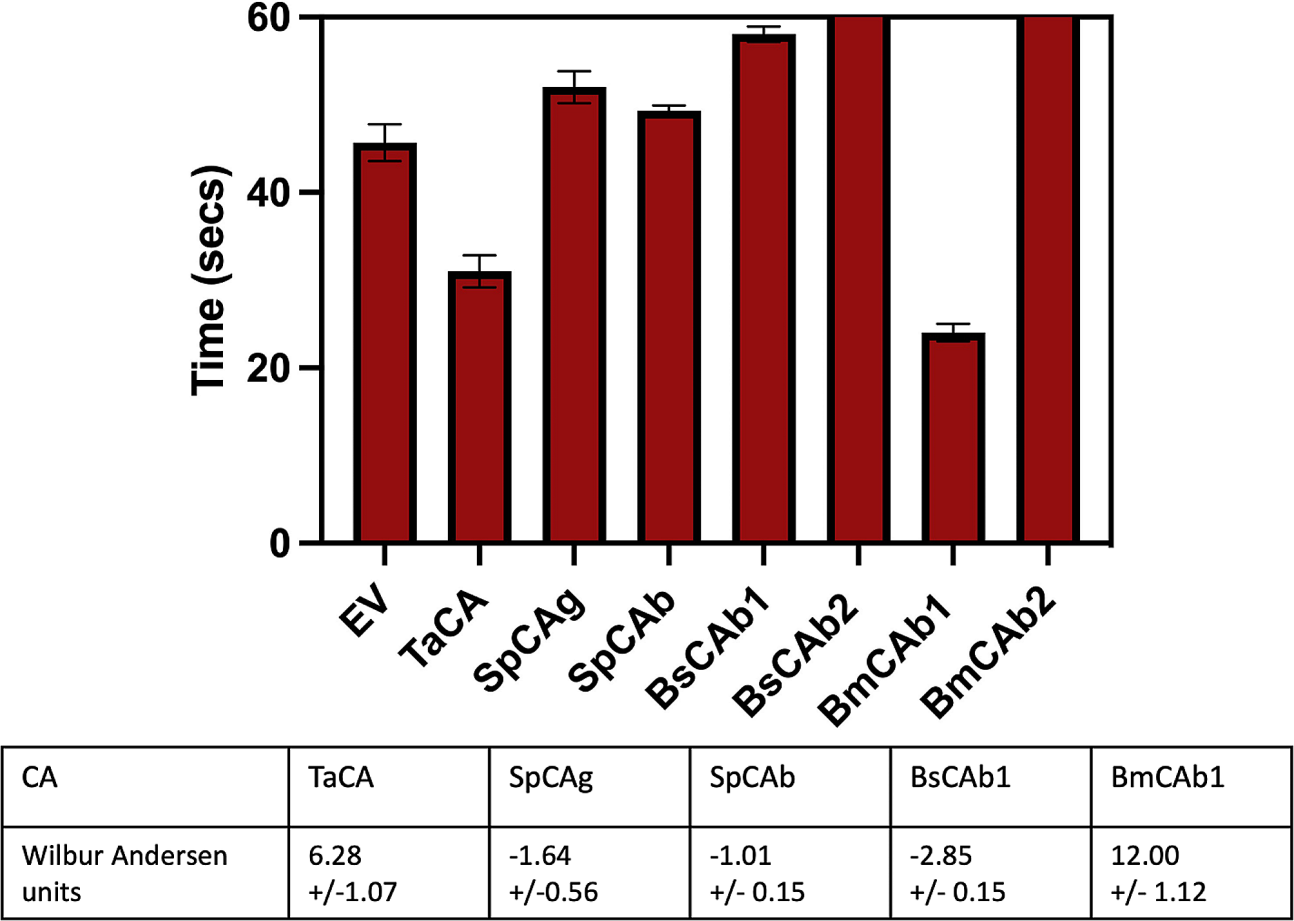
Activity measurements comparing for engineered carbonic anhydrases. The time taken for colour change to occur in the presence of CFE containing CAs (TaCA / BmCAb1 / BmCAb2) and an empty vector (EV) negative control is shown. BsCAb2 and BmCAb2 did not run to completion within 2 min. Error bars show standard error. Table displays Wilbur Andersen units per mg of cell free extract using EV as the control, as BsCAb2 and BmCAb2 did not run to completion, there are no units

CFE from recombinant CAs was assayed for activity using the method adapted from previous publications [14, 22], which uses phenol red to determine how quickly CO_2_ was converted to carbonic acid with the CAs showing significant activity (Fig. 1). A change in the colour of the reaction solution was observed for the CFE of *B. subtilis* transformed with an empty vector (negative control) in just over 40 s. This was expected, considering that *B. subtilis* itself produces CAs with the potential to sequester CO_2_. However, when compared to the negative control, all engineered strains took significantly longer to reach pH 6.6, the point at which phenol red turns from red to yellow, apart from TaCA and BmCAb1, which took a significantly shorter time (*p* = 0.0013 for TaCA and 0.0008 for BmCAb1). When compared to TaCA, BmCAb1 was significantly faster (*p* = 0.0424). In addition, neither BmCAb2 nor BsCAb2 ran to completion during this test (i.e. there was no uniform colour change). However, we cannot simply negate the activities for these two enzymes as the catalysis of CAs for the hydration of CO_2_ is reversible, and depends on the pH of the environment and the active site ionization rate [23, 24]. From these results, Wilbur Anderson units were calculated for the strains which completed the conversion.

The result of negative units for SpCAg, SpCAb and BsCAb1 (Fig. 1) suggests they have little ability to hydrolyse CO_2_. As such, only TaCA, BmCAb1 and BmCAb2 were used for the biomineralization experiments as a high/low activity comparison, with EV alongside *S. pasteurii* and *B. megaterium* as controls.

### MICP through engineered CA strains

Engineered whole cells and CFE for recombinant CAs were used to assess the capability for MICP. For whole cell evaluation, engineered cells were introduced into biocementation media and the expression of CAs was triggered by adding IPTG into the media after the population was established within 24 h growth. *S. pasteurii* and *B. megaterium* was used as positive control and *B. subtilis* with EV was used as negative control For the CFE experiments. CFE derived from *B. subtilis* expressing CAs from a separate culture was added in the same biocementation media.

**Fig. 2:**
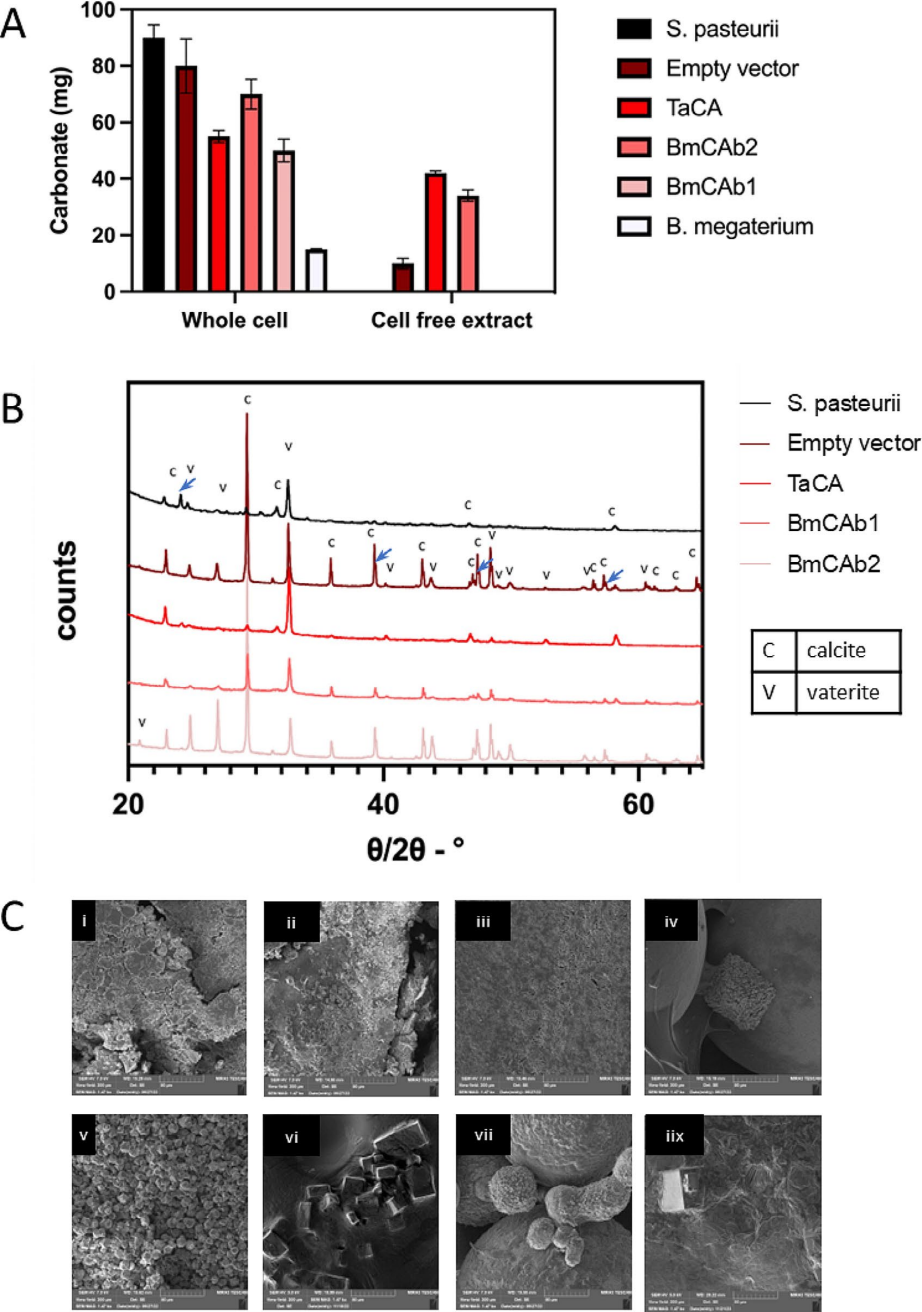
Analysis of carbonate minerals collected from liquid media experiments with engineered carbonic anhydrases. (**A**) mass of minerals recovered from liquid experiments comparing the mineral formation abilities of CAs when used as whole cells and CFEs. Error bars show standard error. (**B**) The XRD spectra of minerals collected from whole cell reactions. Blue arrows point to the spectra for ammonium chloride minerals. (**C**) The corresponding SEM images of minerals from (i) BmCAb1 whole cells, (ii) BmCAb2 whole cells, (iii) TaCA whole cells, (iv) EV whole cells, (v) *S. pasteurii* whole cells (vi) BmCAb2 CFE, (vii) TaCA CFE, (iix) EV CFE

All the whole cell experiments produced measurable amounts of minerals. As expected, *S. pasteurii* yielded the highest amount of minerals, followed by EV and BmCAb2. *B. megaterium* produced the least amount of minerals in this set of experiments (Fig. 2A). In contrast, only EV, TaCA and BmCAb2 CFE resulted in detectable mineral levels, and these were all notably lower than their whole-cell counterparts. This discrepancy is likely due to the absence of cells acting as nucleation sites for crystal formation and growth, an important factor in achieving stable mineral formation during the MICP process [25]. To our surprise, BmCA2 produced larger amount of minerals, both in whole cells and CFE formats although it did not complete the phenol red assay. Equally surprising, despite significant enzyme activity (Fig. 1), the yield of crystals through BmCA1 is significantly lower than the EV control, TaCA and BmCA2. To understand this phenomenon, we further analysed the mineral products through XRD and SEM analysis. Through Reitveld refinement of the XRD analysis [20], it was found that EV samples contained a large amount of ammonium chloride minerals which is present in the media composition (shoulders of labelled carbonate peaks) (Fig. 2B). The ratio of ammonium chloride to carbonate minerals (calcite and vaterite) was 65:35, which, although a high mass, there is in fact less carbonate crystals present in the control than the mass of minerals suggests. TaCA whole cell experiments also contained ammonium chloride, (52% of the crystalline minerals identified), which is likely due to incomplete conversion of available substrate to carbonate. The minerals from MICP reactions using CA containing CFEs were not analysed by XRD due to the low mass recovered.

The SEM images reveal differences in crystal morphology between the whole cell and CFE experiments (Fig. 2C). Experiments with CA containing CFE produced larger crystals (> 25 μm) which appear somewhat oversized, and they are spherical in the case of TaCA containing CFE and rhombohedral in the case of BmCAb2 CFE and EV CFE. Despite the experiment duration being the same, the observation of larger crystals in CFE experiments can be explained by the combination of (1) the availability of the enzyme to interact with the substrates, which would otherwise be separated by a cell membrane and (2) the lack of microbial cells as nucleation sites leads to the growth of existing crystals.

In the case of the whole cell experiments, smaller crystals were formed with the size of 5 μm on average. Samples from *S. pasteurii*, unlike other whole cell experiments, produced relatively unified mineral morphology and suggesting complete conversion to consistently sized and rhombohedral and scalenohedral crystals. The crystalline phase ratio of calcite to vaterite for this control was 73:24, which suggests more favourable conditions for calcite formation over vaterite compared to engineered CA strains, i.e. BmCAb1 (48:52) and BmCAb2 (50:50). Previous studies have demonstrated that the extracellular biopolymers could have significant impact on the morphology and structure of calcium carbonate crystals induced by bacteria [26, 27], we suspect that the different composition of the calcium carbonate crystals we observed here is not only due to the enzyme activities but has also been impacted by the extracellular substances from different bacterial cells, i.e. *B. subtilis* for engineered CAs and *S. pasteurii* for urease. Although both minerals are composed of calcium carbonate, vaterite is a metastable mineral with a hexagonal crystal system and can transition to calcite which is more thermodynamically stable and has a trignomal crystal system [28]. Therefore, for permanent carbon storage, formation of calcite crystals is preferred.

Interestingly, in these reactions, the engineered BmCAb1 whole cell has shown limited ability to produced minerals compared to BmCA2 and even more CFE did not produce any minerals in this set of experiments despite higher enzyme activity (Fig. 1). In addition, a pleasant odour has been recorded along with set of experiments from BmCA2, particularly from CFE reactions, which indicated the volatile compounds have been produced. Previous studies have reported that some CAs also exhibit esterase activities although the mechanism is still not fully clear [29–32]. We suspect BmCA2 potentially belongs to one of those enzymes which requires further analysis.

### CO_2_ sequestration during MICP through engineered systems

**Fig. 3:**
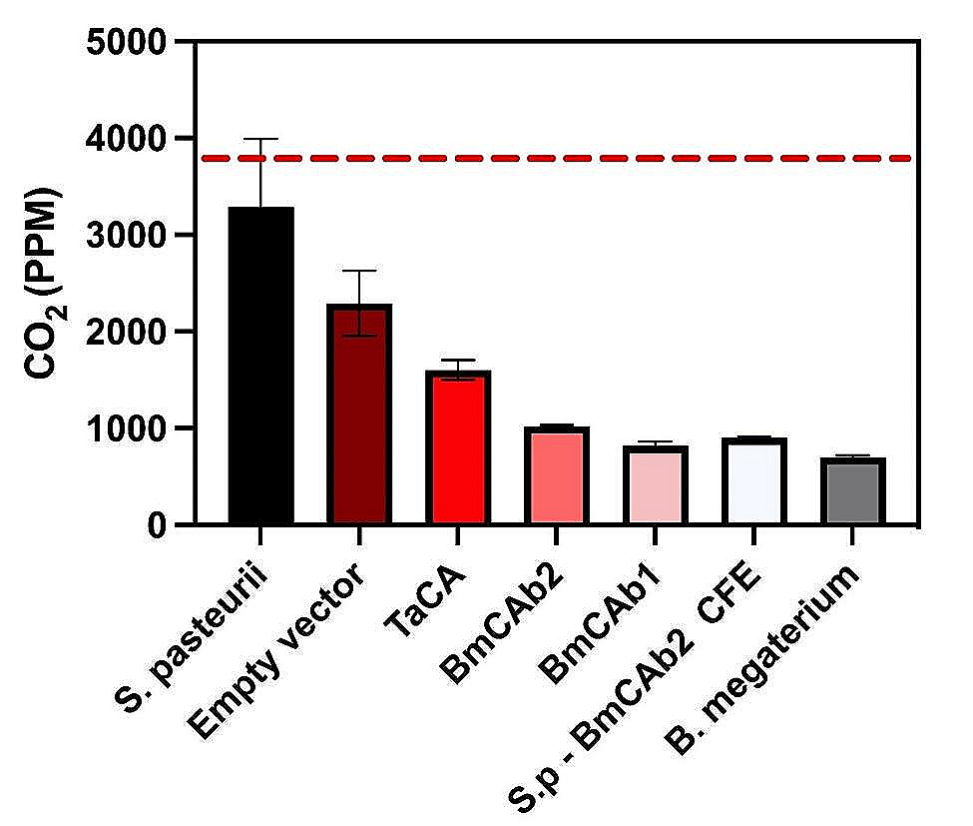
Assessment of CO_2_ sequestration ability on solid media. Final CO_2_ concentration at end of sequestration experiment (54 h post-induction), line represents a baseline with no bacteria present

Unlike urease, carbonic anhydrase is capable of sequestering CO_2_ from the atmosphere and uses this to form bicarbonate [4], which can then be stored as a carbonate mineral. It is this trait that makes CA so attractive for MICP and CO_2_ sequestration at industrial scale. Therefore, it was necessary to test the CO_2_ sequestration abilities of the chosen engineered *B. subtilis*. These were set up on agar plates containing biomineralization nutrient and reactants in the presence of excessive CO_2_ and the concentration of CO_2_ was measured hourly post-induction of CA expression. Compared to a baseline which was measured with no bacteria present, the average CO_2_ concentration was lower throughout the experiments when bacteria were present, including *S. pasteurii* (Figure S2). This observation concurs with other MICP studies using *S. pasteurii* including Okyay & Rodrigues (2015) which demonstrated moderate CO_2_ sequestration [33]. However, comparing with other engineered *B. subtilis* strains, this level is still the highest including *B. subtilis* with EV. The CO_2_ concentrations for all the engineered CAs expressing strains at the termination of the experiment were significantly lower (*p* < 0.0001) compared to EV and *S. pasteurii*. It is noteworthy that, at end of the experiment, both BmCAbs sequestrated a significantly higher level of the CO_2_ compared to well-characterised CA, TaCA with *p* = 0.0035 for BMCAb1 and *p* = 0.0114 for BmCAb2 (Fig. 3) although the BmCAb2 showed little enzyme activity in the colorimetric assay (Fig. 1).

However, when they were compared to *B. megaterium*, where the enzymes initially came from, the CO_2_ concentration was slightly higher at the end of the experiment, and the diffidence is significant (820–1084 PPM compared to 699 PPM, *p* = 0.0487). A similar experiment was set up using *S. pasteurii* as previously described, but with the addition of BmCAb_2_ as a CFE. This set up led to the final concentration of CO_2_ significantly lower than the *S. pasteurii* control (*p* < 0.0001). More interestingly, the mixture sample has sequestrated more CO_2_ level than BmCAb_2_ cells without *S. pasteurii*.

**Fig. 4:**
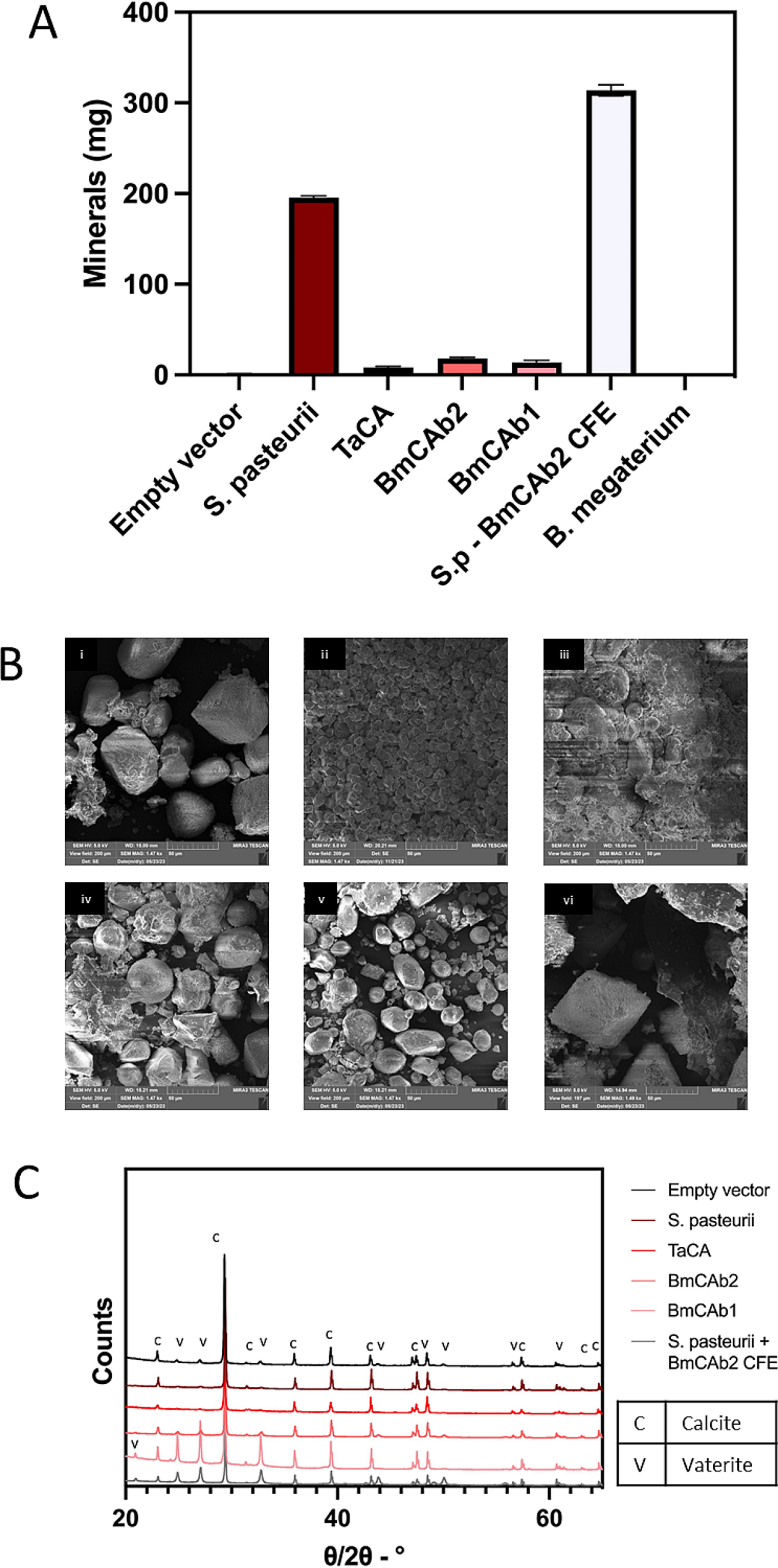
Conversion of CO2 to carbonate by engineered carbonic anhydrases on solid media. (**A**) mass of minerals collected from CO_2_ sequestration experiments; error bars show standard error. (**B**) SEM images of minerals collected from agar plates in CO_2_ sequestration experiment (i) EV control, (ii) positive control *S. pasteurii*, (iii)TaCA, (iv) BmCAb2, (v) BmCAb1, (vi) *S. pasteurii *with BmCAb2 CFE. (C) XRD spectra obtained from minerals recovered, peaks labelled following matches to crystallography open database 2021 database and Rietveld refinement

The minerals precipitated in above CO_2_ sequestration experiments were recovered by boiling to melt the agar. During this process, a small quantity might have been lost due to instability of these minerals at high temperature (approx. 100 °C). The most obvious loss was in the reaction set up with *B. megaterium*. The mass of the stable minerals collected and recorded as shown in Fig. 4A. The largest mass of mineral was collected from the *S. pasteurii* spiked with BmCAb2, (*p* = 0.0087) which was 1.5 folder higher than that of the *S. pasteurii* on its own.

It is likely that the rapid hydrolysis of CO_2_ by CFE is facilitated by the absence of a cell membrane, which otherwise acts as a barrier. The presence of *S. pasteurii* further enhances this process by offering an ideal nucleation point for the conversion of HCO_3_^−^ to stable carbonate minerals, serving as a means of long-term storage. In experiments conducted without *S. pasteurii*, insufficient CO_2_ pressure may have hindered the reversible reaction, resulting in a less favourable environment for continuous HCO_3_^−^ production and mineral precipitation. The synergistic combination of the urease and CA pathways appears to create optimal conditions for efficient CO_2_ capture, followed by its subsequent storage through conversion into calcium carbonate.

The analysis of images obtained by SEM images suggest mature mineral growth in almost all the conditions (Fig. 4B). A mixture of large spherical, smaller rhombohedral and scalenohedral crystals were observed in samples from experiments with *S. pasteurii* (which were particularly small), BmCAb1 and BmCAb2. Large scalenohedral crystals and smaller spherical crystals were observed amongst amorphous debris from minerals collected from experiments with TaCA. Whereas samples collected from experiments with the empty vector control and *S. pasteurii* in combination with CFE containing BmCAb2 were primarily large rhombohedral crystals.

XRD analysis of these minerals found that calcite and vaterite were the only minerals present (Fig. 4C). Unlike XRD from liquid media experiments presented earlier, here there was a larger ratio of calcite compared to vaterite following Rietveld refinement. The calcite crystals in the experiments containing *S. pasteurii*, EV, and TaCA represented 90% of the crystalline phases present, whilst those samples containing BmCAb2 and BmCAb1 were 72% and 48%, respectively. The experiments set up with BmCAb2 CFE plus *S. pasteurii* yield the least calcite with 46%. A possible explanation for the lower proportion of calcite present in this experiment is that there were significant combined enzyme activities from both urea hydrolysis and CO_2_ hydration led to rapid precipitation at a high supersaturation level which yield large amount less thermodynamically stable form of CaCO_3_, i.e. vaterites observed in this experiment. However, a previous study demonstrated that this generally could change into a more stable phase at a particular condition [34, 35] therefore, we suspect that the vaterite crystals would have advanced to calcite given more time for this experiment. The techniques presented here did allow for semi-quantification of the crystalline minerals formed, however, it should be noted that any amorphous crystals were not identified and could have an impact on the properties of the bulk material [36].

Similarly, an experiment measuring bacillus produced carbonic anhydrase also identified calcite through XRD as the end carbonate mineral produced in conjunction with a decrease in CO_2_ [37] resulting in a maximum of 1.8 g of mineral produced from a 250 mL culture. Although of greater mass than achieved here, our culture size was smaller and our mass per ml was much larger for those which combined *S. pasteurii* and CA.

Although the solid media samples were grown for less time than those in liquid media, there was a higher mass of mineral produced, of a more stable nature (more calcite than vaterite) and larger mineral structures formed. Whilst TaCA did generate as high a mass of minerals as BmCAs, the structure of TaCA is well understood [14] and a more in-depth characterization of the kinetics and structure of BmCAs would aid in understanding why they appear to sequester more CO_2_ over time and explain the difference in time scales between CAs as seen in the phenol red assay. This information may also go some way to explaining the differences in size and type of crystals formed. Large calcite crystals, such as those produced by *S. pasteurii* with BmCAb2 are considered more stable than smaller crystals or vaterite. As vaterite is a transitional mineral [38], it is possible that a greater proportion would progress to calcite if this experiment were prolonged. In addition to the enzyme activity, the morphology was also influenced by the other molecules in the environment such as extracellular polymeric substance in the whole cell experiments and other molecules such as proteins and polysaccharides in the CFEs. Therefore, differences in morphology as observed in BmCA2 whole cell, CFE, and in agar, could be, in part, due to these factors.

BmCAb1 and BmCAb2 have been shown to be effective CAs capable of capturing CO_2_ and storing it as carbonate materials when compared to previously characterized highly active CA (TaCA). They have been shown to function in both a CFE and within the cell. One downside of CAs is the possibility of allowing the reverse reaction to occur, with an excess of CO_2_ required to drive the reaction towards carbon capture. In our experiments an increase in CO_2_ concentration may help to drive this reaction towards HCO_3_^−^ production [39], and for this reason industrial use of CAs may be most suited to areas of high CO_2_ production such as steel works.

To understand how MICP via BmCAb2 could occur in a biocementation setting, further tests in geoengineering or other environments more similar to real-world applications are necessary. The media used in these experiments as both liquid and agar contains high concentrations of the substrates necessary for MICP and a lower concentration of nutrient broth (compared to standard laboratory practice), however this is still greater than would be expected in environmental settings and it has been shown that media composition can significantly affect CO_2_ sequestration by CA [40]. Furthermore, the scale of the experiments presented here are far smaller than would be expected in industrial applications and so would have to be considered. For these reasons, a life cycle assessment would be necessary to ensure this technology is sustainable at scale.

Another possible solution is ensuring the correct environment for calcite precipitation to occur is always available, which could be achieved by inclusion of urease. Other attempts to introduce ureolytic bacteria to soil samples as a biocementation tool have found that both *Bacillus* and *Sporosarcina* remain present within soil samples [41] but suggests utilizing a feedstock of bacteria already primed for MICP may be the most effective option. Here, we have demonstrated that combining heterogeneous CA with urease producing bacteria could lead to higher level of CO_2_ sequestration and CaCO_3_ precipitation. Therefore, we believe that an engineered *B. subtilis* producing CA could be used commercially in place of CAs currently being used by industries such as CO_2_ Solutions (Canada) [9], and Carbozyme (United States) [12] to generate effective carbon capture and storage in high CO_2_ environments. Previous studies have demonstrated that there is a synergistic relationship between urease and CA [42], however, they have not engineered strains to control the expression of these enzymes or considered the CO_2_ sequestering ability of such a system.

In this study it is considered that BmCAb2 would be more suitable than BmCAb1 due to its ability to produce minerals in both a CFE and within a cell, and the increased size of the minerals formed compared to BmCAb1. This longer time taken to hydrate CO_2_ during the phenol red assay indicates a lower activity, providing a possible reason for the increase in size.

By combining CA and urease, a fast and effective carbon capture and storage system can be established. *B. megaterium* has both these enzymes [16] and has been explored in various geoengineering and material engineering applications [43, 44]. However, as shown in this study, wild type *B. megaterium* did not display the ability to store carbon as a calcium carbonate mineral. This finding contradicts some of other studies which have shown *B. megaterium* to outperform *S. pasteurii* in terms of producing carbonate minerals [45, 46], but it is notable that experiments conducted in prior literature were performed at low temperatures (15 °C), whereas our experiments were set up at 30 °C. In fact, Sun et al. demonstrated that the precipitation rate for *S. pasteurii* is higher than *B. megaterium* at 30 °C [44]. Likewise, *B. subtilis* does have its own native CA [47] and has been shown to improve soil stability as non-ureolytic bacteria [48]. However, as evidenced in the EV control, it is not as effective as the engineered strains or other controls, albeit this may in part be due to the basal expression of these CAs compared to overexpression of BmCAb2 and BmCAb1.

Conversely, *S. pasteurii* is known to be an excellent organism to promote carbonate precipitation, but as seen here, the CO_2_ sequestration as part of this process is minimal. Combining the CO_2_ capturing abilities of BmCAb2 and the carbon storage abilities of *S. pasteurii*, a system by which biocementation occurs while synchronously lowering the CO_2_ concentration of the atmosphere may be possible as opposed to current cement production which contributes substantial CO_2_ to global emissions [1]. Previously, it was shown that a synergistic relationship occurs between these two processes [42, 49, 50] whereby the urease pathway maintains the correct conditions for carbonate mineral precipitation and CA creates an excess of bicarbonate ions. Although this work was carried out in the context of generating cementitious material, this technology could also be developed for use in ground improvement to build on previous work which found that native *Bacillus cereus* producing CA could improve sand curing [51]. CA has also been shown to be effective in bioremediation of steel slag [52], and utilisation of these novel strains in conjunction with *S. pasteurii* could be used in the same way.

## Conclusion

Novel CAs were successfully recombinantly expressed in *B. subtilis* and could sequester CO_2_ to produce calcium carbonate minerals. This production was increased when CFE containing CA was used in conjunction was *S. pasteurii*, with no loss of CO_2_ sequestering ability. By employing the biological engineering approach outlined here, a framework for comprehending an optimized pathway for CO_2_ sequestration coupled with mineral production can be initiated. Further exploration is warranted to elucidate the most cost-effective strategies for achieving this goal, whether through the co-cultivation of our engineered *B. subtilis* with *S. pasteurii* or by direct engineering of *S. pasteurii* itself.

## Data Availability

All data generated or analysed during this study are included in this manuscript. Further data including source data are available from the corresponding author on reasonable request.

## Abbreviations

2YT: 2X Yeast Tryptone
ANOVA: Analysis of variance
CA: Carbonic anhydrase
CFE: Cell free extract
CO_2_: Carbon dioxide
EPRI: Electric power research institute
EV: Empty vector
IPTG: Isopropyl-β-d-1-thiogalactopyranoside
LB: Luria Broth
MICP: Microbially induced calcium carbonate precipitation
SEM: Scanning electron microscopy
XRD: X-ray diffraction
